# (*Z*)-1-[2-(Trifluoro­meth­yl)benzyl­idene]thio­semicarbazide

**DOI:** 10.1107/S1600536811047623

**Published:** 2011-11-19

**Authors:** Xin Chen, Zuo-Liang Jing

**Affiliations:** a College of Sciences, Tianjin University of Science and Technology, Tianjin 300222, People’s Republic of China

## Abstract

In the crystal structure of the title compound, C_9_H_8_F_3_N_3_S, all atoms except for two of the F atoms are located on a mirror plane. In the crystal, the molecules are connected by N—H⋯S hydrogen bonds, forming a mol­ecular tape along the *a* axis.

## Related literature

For general background to metal complexes with Shiff bases, see: Kahwa *et al.* (1986 ▶); Deng *et al.* (2005 ▶). For related structures, see: Guo *et al.* (2006 ▶); Jing *et al.* (2005 ▶); Santos *et al.* (2001 ▶); Yu *et al.* (2005 ▶).
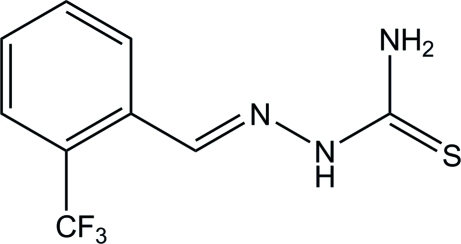


## Experimental

### 

#### Crystal data

C_9_H_8_F_3_N_3_S
*M* *_r_* = 247.24
Orthorhombic, 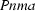

*a* = 8.628 (2) Å
*b* = 6.9795 (17) Å
*c* = 18.222 (4) Å
*V* = 1097.2 (5) Å^3^
*Z* = 4
Mo *K*α radiation
μ = 0.31 mm^−1^
*T* = 294 K
0.40 × 0.40 × 0.30 mm

#### Data collection

Bruker SMART APEX CCD area-detector diffractometer
Absorption correction: multi-scan (*SADABS*; Sheldrick, 1996 ▶) *T*_min_ = 0.886, *T*_max_ = 0.912
5953 measured reflections
1228 independent reflections
869 reflections with *I* > 2σ(*I*)
*R*_int_ = 0.028

#### Refinement

*R*[*F* ^2^ > 2σ(*F* ^2^)] = 0.036
*wR*(*F* ^2^) = 0.113
*S* = 1.07
1228 reflections
101 parameters
H atoms treated by a mixture of independent and constrained refinement
Δρ_max_ = 0.19 e Å^−3^
Δρ_min_ = −0.20 e Å^−3^

### 

Data collection: *SMART* (Bruker, 1999 ▶); cell refinement: *SAINT* (Bruker, 1999 ▶); data reduction: *SAINT*; program(s) used to solve structure: *SHELXS97* (Sheldrick, 2008 ▶); program(s) used to refine structure: *SHELXL97* (Sheldrick, 2008 ▶); molecular graphics: *SHELXTL* (Sheldrick, 2008 ▶); software used to prepare material for publication: *SHELXTL*.

## Supplementary Material

Crystal structure: contains datablock(s) I, global. DOI: 10.1107/S1600536811047623/is2791sup1.cif

Structure factors: contains datablock(s) I. DOI: 10.1107/S1600536811047623/is2791Isup2.hkl

Supplementary material file. DOI: 10.1107/S1600536811047623/is2791Isup3.cml

Additional supplementary materials:  crystallographic information; 3D view; checkCIF report

## Figures and Tables

**Table 1:** Hydrogen-bond geometry (Å, °)

*D*—H⋯*A*	*D*—H	H⋯*A*	*D*⋯*A*	*D*—H⋯*A*
N3—H3*B*⋯S1^i^	0.88 (4)	2.54 (4)	3.418 (3)	174 (3)
N2—H2*A*⋯S1^ii^	0.82 (3)	2.61 (3)	3.430 (2)	173 (3)

Symmetry codes: (i) 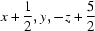; (ii) 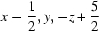.

**Fig. 1.**
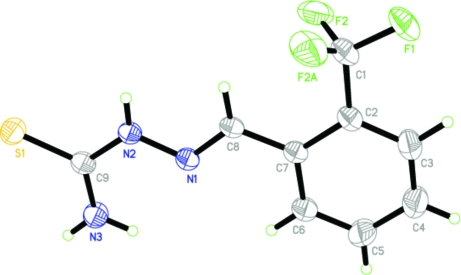
The molecular structure of the title compound, with displacement ellipsoids drawn at the 30% probability level.

**Fig. 2.**
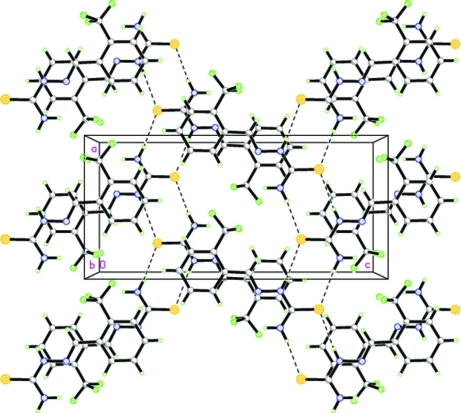
A packing diagram of the title compound viewed down the b axis, showing intermolecular hydrogen bonds (dashed lines).

**Fig. 3.**
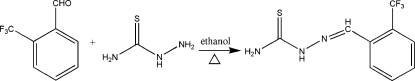
The synthetic scheme of the title compound.

## supplementary crystallographic information

### Comment

Metal complexes based on Schiff bases have attracted much attention because they can be utilized as model compounds of active centres in various proteins and enzymes (Kahwa *et al.*, 1986; Santos *et al.*, 2001). As part of an investigation of the coordination properties of Shiff bases functioning as ligands (Yu *et al.*, 2005; Deng *et al.*, 2005; Jing, Fan *et al.*, 2005; Guo, Sun *et al.*, 2006), we report the synthesis and structure of the title compound, (I). In the molecular structure of the title compound (Fig. 1), the expected geometric parameters are observed. The molecules are associated *via* weak intermolecular N—H···S hydrogen-bonding interactions (Table 1) to form a supramolecular network as illustrated in Fig. 2.

### Experimental

An anhydrous ethanol solution(50 mL) of thiosemicarbazide (0.91 g, 10 mmol) was added to an anhydrous ethanol solution(50 mL) of 2-(trifluoromethyl)benzaldehyde (1.74 g, 10 mmol) and the mixture was stirred at 350 K for 6 h under N_2_, whereupon a straw colorless solution appeared. The solvent was removed and the residue recrystallized from anhydrous ethanol. The product was isolated and then dried *in vacuo* to give pure (I) in 77% yield (Fig. 3). The colorless single crystals suitable for X-ray analysis were obtained by slow evaporation of an anhydrous ethanol solution of (I).

### Refinement

The N-bound H atoms were located in a difference Fourier map and their positions were refined freely with *U*_iso_(H) = 1.2*U*_eq_(N) [N—H = 0.82 (3)–0.93 (4) Å]. C-bound H atoms were included in calculated positions (C—H = 0.93 Å) and refined using the riding model approximation, with *U*_iso_(H) = 1.2*U*_eq_(C).

### Crystal data

**Table:**

C_9_H_8_F_3_N_3_S	*F*(000) = 504
*M**_r_* = 247.24	*D*_x_ = 1.497 Mg m^−^^3^
Orthorhombic, *P**n**m**a*	Mo *K*α radiation, λ = 0.71073 Å
Hall symbol: -P 2ac 2n	Cell parameters from 2111 reflections
*a* = 8.628 (2) Å	θ = 2.6–26.2°
*b* = 6.9795 (17) Å	µ = 0.31 mm^−^^1^
*c* = 18.222 (4) Å	*T* = 294 K
*V* = 1097.2 (5) Å^3^	Block, colorless
*Z* = 4	0.40 × 0.40 × 0.30 mm

### Data collection

**Table:**

Bruker SMART APEX CCD area-detector diffractometer	1228 independent reflections
Radiation source: fine-focus sealed tube	869 reflections with *I* > 2σ(*I*)
graphite	*R*_int_ = 0.028
Detector resolution: 7.31 pixels mm^-1^	θ_max_ = 26.4°, θ_min_ = 2.2°
phi and ω scans	*h* = −10→7
Absorption correction: multi-scan (*SADABS*; Sheldrick, 1996)	*k* = −7→8
*T*_min_ = 0.886, *T*_max_ = 0.912	*l* = −22→22
5953 measured reflections	

### Refinement

**Table:**

Refinement on *F*^2^	Secondary atom site location: difference Fourier map
Least-squares matrix: full	Hydrogen site location: inferred from neighbouring sites
*R*[*F*^2^ > 2σ(*F*^2^)] = 0.036	H atoms treated by a mixture of independent and constrained refinement
*wR*(*F*^2^) = 0.113	*w* = 1/[σ^2^(*F*_o_^2^) + (0.0497*P*)^2^ + 0.3737*P*] where *P* = (*F*_o_^2^ + 2*F*_c_^2^)/3
*S* = 1.07	(Δ/σ)_max_ = 0.001
1228 reflections	Δρ_max_ = 0.19 e Å^−^^3^
101 parameters	Δρ_min_ = −0.20 e Å^−^^3^
0 restraints	Extinction correction: *SHELXL97* (Sheldrick, 2008), Fc^*^=kFc[1+0.001xFc^2^λ^3^/sin(2θ)]^-1/4^
Primary atom site location: structure-invariant direct methods	Extinction coefficient: 0.0104 (19)

### Special details

**Table:**

Geometry. All e.s.d.'s (except the e.s.d. in the dihedral angle between two l.s. planes) are estimated using the full covariance matrix. The cell e.s.d.'s are taken into account individually in the estimation of e.s.d.'s in distances, angles and torsion angles; correlations between e.s.d.'s in cell parameters are only used when they are defined by crystal symmetry. An approximate (isotropic) treatment of cell e.s.d.'s is used for estimating e.s.d.'s involving l.s. planes.
Refinement. Refinement of *F*^2^ against ALL reflections. The weighted *R*-factor *wR* and goodness of fit *S* are based on *F*^2^, conventional *R*-factors *R* are based on *F*, with *F* set to zero for negative *F*^2^. The threshold expression of *F*^2^ > σ(*F*^2^) is used only for calculating *R*-factors(gt) *etc*. and is not relevant to the choice of reflections for refinement. *R*-factors based on *F*^2^ are statistically about twice as large as those based on *F*, and *R*- factors based on ALL data will be even larger.

### Fractional atomic coordinates and isotropic or equivalent isotropic displacement parameters (Å^2^)

**Table:**

	*x*	*y*	*z*	*U*_iso_*/*U*_eq_	
S1	0.72882 (8)	0.2500	1.28034 (4)	0.0803 (4)	
F1	0.0437 (2)	0.2500	0.91939 (12)	0.0999 (8)	
F2	0.15033 (14)	0.0964 (2)	1.00795 (8)	0.0891 (5)	
N1	0.6005 (2)	0.2500	1.07458 (11)	0.0465 (6)	
N2	0.5981 (2)	0.2500	1.14995 (12)	0.0519 (6)	
H2A	0.513 (4)	0.2500	1.1704 (16)	0.062*	
N3	0.8620 (3)	0.2500	1.14879 (15)	0.0726 (9)	
H3A	0.862 (4)	0.2500	1.098 (2)	0.087*	
H3B	0.953 (5)	0.2500	1.1704 (19)	0.087*	
C1	0.1665 (3)	0.2500	0.96416 (18)	0.0636 (8)	
C2	0.3177 (3)	0.2500	0.92383 (15)	0.0490 (7)	
C3	0.3171 (4)	0.2500	0.84755 (17)	0.0691 (9)	
H3	0.2231	0.2500	0.8226	0.083*	
C4	0.4530 (4)	0.2500	0.80832 (18)	0.0847 (11)	
H4	0.4510	0.2500	0.7573	0.102*	
C5	0.5928 (4)	0.2500	0.84529 (17)	0.0775 (10)	
H5	0.6851	0.2500	0.8189	0.093*	
C6	0.5970 (3)	0.2500	0.92107 (16)	0.0561 (8)	
H6	0.6922	0.2500	0.9451	0.067*	
C7	0.4604 (3)	0.2500	0.96217 (14)	0.0428 (6)	
C8	0.4682 (3)	0.2500	1.04285 (14)	0.0442 (6)	
H8	0.3777	0.2500	1.0706	0.053*	
C9	0.7324 (3)	0.2500	1.18753 (16)	0.0538 (7)	

### Atomic displacement parameters (Å^2^)

**Table:**

	*U*^11^	*U*^22^	*U*^33^	*U*^12^	*U*^13^	*U*^23^
S1	0.0329 (4)	0.1575 (10)	0.0504 (4)	0.000	−0.0050 (3)	0.000
F1	0.0416 (10)	0.161 (2)	0.0973 (15)	0.000	−0.0225 (9)	0.000
F2	0.0515 (7)	0.1089 (12)	0.1070 (11)	−0.0201 (7)	0.0044 (7)	0.0278 (10)
N1	0.0349 (11)	0.0577 (15)	0.0469 (12)	0.000	0.0003 (9)	0.000
N2	0.0282 (11)	0.0807 (18)	0.0468 (12)	0.000	0.0005 (9)	0.000
N3	0.0300 (11)	0.132 (3)	0.0555 (14)	0.000	−0.0005 (11)	0.000
C1	0.0383 (14)	0.082 (2)	0.071 (2)	0.000	−0.0100 (13)	0.000
C2	0.0419 (13)	0.0487 (16)	0.0565 (15)	0.000	−0.0049 (12)	0.000
C3	0.0595 (18)	0.091 (3)	0.0566 (17)	0.000	−0.0154 (14)	0.000
C4	0.074 (2)	0.129 (3)	0.0511 (17)	0.000	−0.0017 (17)	0.000
C5	0.062 (2)	0.113 (3)	0.0578 (18)	0.000	0.0124 (15)	0.000
C6	0.0410 (14)	0.069 (2)	0.0587 (16)	0.000	0.0015 (12)	0.000
C7	0.0381 (13)	0.0409 (15)	0.0495 (14)	0.000	−0.0003 (11)	0.000
C8	0.0305 (12)	0.0507 (16)	0.0513 (14)	0.000	0.0019 (10)	0.000
C9	0.0321 (12)	0.074 (2)	0.0554 (15)	0.000	−0.0012 (11)	0.000

### Geometric parameters (Å, °)

**Table:**

S1—C9	1.691 (3)	C2—C3	1.390 (4)
F1—C1	1.338 (3)	C2—C7	1.416 (3)
F2—C1	1.343 (2)	C3—C4	1.373 (5)
N1—C8	1.279 (3)	C3—H3	0.9300
N1—N2	1.374 (3)	C4—C5	1.381 (5)
N2—C9	1.346 (3)	C4—H4	0.9300
N2—H2A	0.82 (3)	C5—C6	1.381 (4)
N3—C9	1.322 (3)	C5—H5	0.9300
N3—H3A	0.93 (4)	C6—C7	1.397 (4)
N3—H3B	0.88 (4)	C6—H6	0.9300
C1—F2^i^	1.343 (2)	C7—C8	1.472 (3)
C1—C2	1.497 (4)	C8—H8	0.9300
			
C8—N1—N2	116.0 (2)	C3—C4—C5	119.4 (3)
C9—N2—N1	119.7 (2)	C3—C4—H4	120.3
C9—N2—H2A	122 (2)	C5—C4—H4	120.3
N1—N2—H2A	118 (2)	C6—C5—C4	120.7 (3)
C9—N3—H3A	122 (2)	C6—C5—H5	119.7
C9—N3—H3B	121 (2)	C4—C5—H5	119.7
H3A—N3—H3B	117 (3)	C5—C6—C7	120.9 (3)
F1—C1—F2^i^	106.24 (16)	C5—C6—H6	119.5
F1—C1—F2	106.24 (16)	C7—C6—H6	119.5
F2^i^—C1—F2	105.8 (3)	C6—C7—C2	118.0 (2)
F1—C1—C2	113.0 (3)	C6—C7—C8	119.8 (2)
F2^i^—C1—C2	112.47 (15)	C2—C7—C8	122.2 (2)
F2—C1—C2	112.47 (15)	N1—C8—C7	119.5 (2)
C3—C2—C7	119.8 (3)	N1—C8—H8	120.3
C3—C2—C1	119.2 (3)	C7—C8—H8	120.3
C7—C2—C1	121.0 (2)	N3—C9—N2	117.1 (2)
C4—C3—C2	121.2 (3)	N3—C9—S1	123.3 (2)
C4—C3—H3	119.4	N2—C9—S1	119.5 (2)
C2—C3—H3	119.4		
			
C8—N1—N2—C9	180.000 (1)	C5—C6—C7—C2	0.000 (2)
F1—C1—C2—C3	0.000 (2)	C5—C6—C7—C8	180.000 (1)
F2^i^—C1—C2—C3	−120.30 (18)	C3—C2—C7—C6	0.000 (2)
F2—C1—C2—C3	120.30 (18)	C1—C2—C7—C6	180.000 (1)
F1—C1—C2—C7	180.000 (1)	C3—C2—C7—C8	180.000 (1)
F2^i^—C1—C2—C7	59.70 (18)	C1—C2—C7—C8	0.000 (1)
F2—C1—C2—C7	−59.70 (18)	N2—N1—C8—C7	180.000 (1)
C7—C2—C3—C4	0.000 (2)	C6—C7—C8—N1	0.000 (1)
C1—C2—C3—C4	180.000 (1)	C2—C7—C8—N1	180.000 (1)
C2—C3—C4—C5	0.000 (2)	N1—N2—C9—N3	0.000 (2)
C3—C4—C5—C6	0.000 (2)	N1—N2—C9—S1	180.0
C4—C5—C6—C7	0.000 (2)		

Symmetry codes: (i) *x*, −*y*+1/2, *z*.

### Hydrogen-bond geometry (Å, °)

**Table:**

*D*—H···*A*	*D*—H	H···*A*	*D*···*A*	*D*—H···*A*
N3—H3B···S1^ii^	0.88 (4)	2.54 (4)	3.418 (3)	174 (3)
N2—H2A···S1^iii^	0.82 (3)	2.61 (3)	3.430 (2)	173 (3)

Symmetry codes: (ii) *x*+1/2, *y*, −*z*+5/2; (iii) *x*−1/2, *y*, −*z*+5/2.
